# Effects of arch support doses on the center of pressure and pressure distribution of running using statistical parametric mapping

**DOI:** 10.3389/fbioe.2022.1051747

**Published:** 2022-11-21

**Authors:** Jiale Cheng, Qing Zeng, Jiaqi Lai, Xianyi Zhang

**Affiliations:** ^1^ School of Biomedical Engineering, Shenzhen Campus of Sun Yat-sen University, Shenzhen, China; ^2^ Key Laboratory of Sensing Technology and Biomedical Instrument of Guangdong Province, School of Biomedical Engineering, Sun Yat-sen University, Guangzhou, China; ^3^ Department of Rehabilitation Medicine, Zhujiang Hospital, Southern Medical University, Guangzhou, China; ^4^ School of Rehabilitation Medicine, Southern Medical University, Guangzhou, China

**Keywords:** center of pressure, pressure distribution, statistical parametric mapping, arch support, running biomechanics

## Abstract

Insoles with an arch support have been used to address biomechanical risk factors of running. However, the relationship between the dose of support and running biomechanics remains unclear. The purpose of this study was to determine the effects of changing arch support doses on the center of pressure (COP) and pressure mapping using statistical parametric mapping (SPM). Nine arch support variations (3 heights * 3 widths) and a flat insole control were tested on fifteen healthy recreational runners using a 1-m Footscan pressure plate. The medial-lateral COP (COP_ML_) coordinates and the total COP velocity (COPV_total_) were calculated throughout the entirety of stance. One-dimensional and two-dimensional SPM were performed to assess differences between the arch support and control conditions for time series of COP variables and pressure mapping at a pixel level, respectively. Two-way ANOVAs were performed to test the main effect of the arch support height and width, and their interaction on the peak values of the COPV_total_. The results showed that the COPV_total_ during the forefoot contact and forefoot push off phases was increased by arch supports, while the COP medial-lateral coordinates remained unchanged. There was a dose-response effect of the arch support height on peak values of the COPV_total_, with a higher support increasing the first and third valleys but decreasing the third peak of the COPV_total_. Meanwhile, a higher arch support height shifted the peak pressure from the medial forefoot and rearfoot to the medial arch. It is concluded that changing arch support doses, primarily the height, systematically altered the COP velocities and peak plantar pressure at a pixel level during running. When assessing subtle modifications in the arch support, the COP velocity was a more sensitive variable than COP coordinates. SPM provides a high-resolution view of pressure comparisons, and is recommended for future insole/footwear investigations to better understand the underlying mechanisms and improve insole design.

## 1 Introduction

Running-related injuries primarily result from an accumulation of repetitive stress applied to the body, and technical improvements (e.g., footwear and insoles) could help address biomechanical risk factors such as controlling joint motions and shifting musculoskeletal loading (Willwacher et al., 2022). Among footwear/insole designs, the arch support has been frequently adopted to manage lower limb complaints by improving the dynamic function of lower limbs (Hunt et al., 2017; Peng et al., 2021). Foot posture with a lower arch has been associated with lower limb pathologies including exercise-related injuries, medial tibial stress syndrome (MTSS) and patellofemoral pain (Menz et al., 2013; Neal et al., 2014). Compared with a flat insole control, using an arch support significantly reduces the impact after strike, redistributes plantar pressure and improves lower limb coordination (Fong et al., 2020; Huang et al., 2020; Jafarnezhadgero et al., 2020).

The human foot arch deforms during gait to fulfill its role in storing and recoiling elastic energy and acting as a lever during push-off (Stearne et al., 2016). The amount of arch support determines the allowed arch deformation. To optimize arch support parameters for running, it is essential to establish the dose-response relationship between arch support parameters and running biomechanics. Although the human foot arch deforms in a 3D manner, previous studies mainly evaluated the effect of arch support height alterations on biomechanical risk factors of running-related injuries, such as rearfoot kinematics (Wahmkow et al., 2017) and the center of pressure (COP) medial-lateral displacements during running (Zhang et al., 2022). However, no systematic effects on these biomechanical variables were observed by changing the arch support parameters. From mechanical perspective, changing the doses of the arch support would induce corresponding alterations in running biomechanics at some level. A more sensitive biomechanical variable that changes systematically with the arch support parameter is warranted.

The plantar pressure variables, including the COP and plantar pressure mapping, are subject measures of foot function during gait and excessive pressure on foot tissues have been associated with a higher risk of running-related injuries (De Cock et al., 2008; Fernández-Seguín et al., 2014; Bergstra et al., 2015). There are also other approaches to analyze plantar pressure patterns. For instance, entropy has been used to analyze the complexity of plantar pressure patterns (Liau et al., 2019; Liau et al., 2021). Several experimental studies have shown that plantar pressure variables were sensitive to variations in the rearfoot and forefoot components of the insole. Telfer et al. (2013a) found a linear dose-response effect of rearfoot wedges on the peak pressure of midfoot and lateral rearfoot during running. Zhang et al. (2022) showed a linear dose-response effect of forefoot wedges on the force-time integral under hallux and on the COP medial-lateral displacements during propulsion of running. The COP velocity during the forefoot contact phase of walking was also sensitive to the rearfoot elevation of insoles (Zhang and Li, 2014). It is, therefore, promising to find a systematic relationship between the arch support doses and pressure variables during running using experimental approaches.

It needs to be noted that most previous studies employed traditional subsampling approach to analyze the COP and pressure mapping (Xu et al., 2019; Duan et al., 2022; Xiang et al., 2022), which usually calculates the mean and maximum values of pressure data and divides the foot into a small number of zones. For instance, the mean value of the COP velocity of sub-phases of gait has been used to evaluate gait stability (Zhang and Li, 2014). The foot has been divided into 6 to 10 anatomical regions, and the mean pressure variables of those regions were compared with and without the use of the arch support (Farzadi et al., 2015; Naderi et al., 2019; Fong et al., 2020; Huang et al., 2020). Although this method can effectively reduce the large data set to a manageable size, ignoring most of the data may lead to inaccurate results, potentially leading to misinterpretations of foot function (Pataky and Goulermas, 2008). A systematic review suggested that contradictory results on pressure variables were reported in studies using different methods of subdividing the plantar area (Mann et al., 2016).

Alternatively, statistical parametric mapping (SPM), which originated in the field of neuroimaging (Friston et al., 1994), has been used to analyze one-dimensional (1D) and two-dimensional (2D) data in a continuous manner (Pataky and Goulermas, 2008). This process allows comparisons in more regions of interest, and reduces the likelihood of discarding potentially relevant information (Pataky et al., 2013). Hence, it has been increasing adopted in analyzing cyclical signals, such as cyclical joint angles (Pataky et al., 2013; D’Isidoro et al., 2020) and muscle activity patterns (Nuesch et al., 2019). 1D SPM can analyze time series data, such as the COP trajectories over the stance phase, at each sampling point in the time domain. 2D SPM works by bringing the plantar pressure images from all participants into anatomical correspondence, performing statistical tests at each sample point in the space domain (Booth et al., 2018). Based on a General Linear Model of the data analyses each pixel using standard (univariate) statistical tests under the null hypothesis. This method can generate continuous statistical maps across the entire plantar foot and therefore can detect local pressure alterations at a higher spatial resolution. As hypothesis testing in a continuous manner could reduce post hoc regional focus bias (Pataky et al., 2011), using SPM may help detect biomechanical variables that are sensitive to subtle insole modification.

The purpose of this study was to determine the acute effect of arch supports varying in the height and width on the COP coordinates, COP velocities and plantar pressure mapping of running using SPM. Nine variations of arch supports (3 heights * 3 widths) were designed based on the morphological changes of the arch under different weight bearing conditions. This study hypothesized that using an arch support would alter the COP variables and plantar pressure mapping, and more specifically, that a higher and wider arch support would induce a lateral shift of the COP, a faster COP advancement during push-off, and higher plantar pressure under the medial midfoot.

## 2 Materials and methods

**FIGURE 1:**
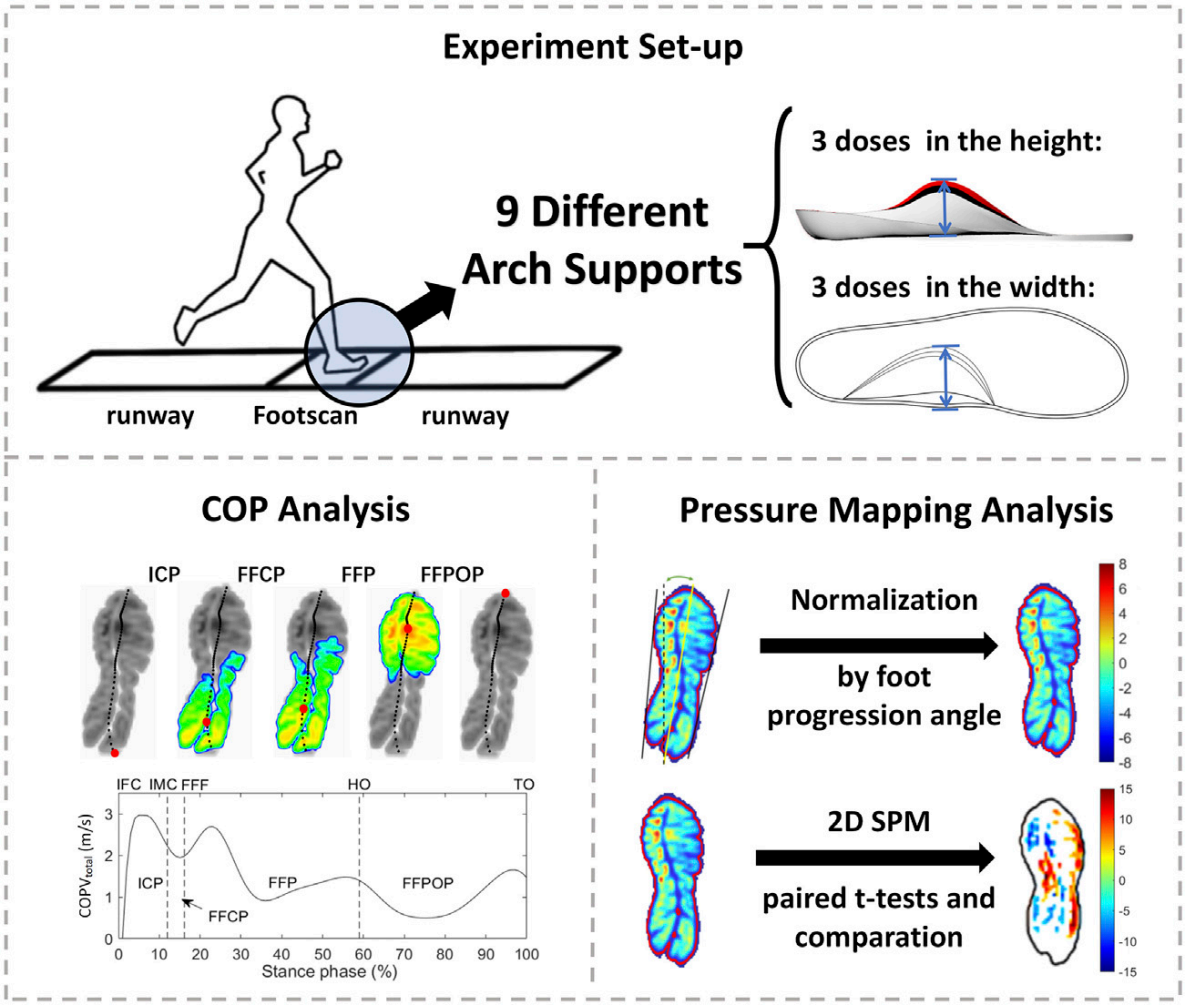
The experimental setting. ICP, initial contact phase; FFCP, forefoot contact phase; FFP, foot flat phase; FFPOP, forefoot push off phase; IFC, initial foot contact; IMC, initial metatarsal contact; FFC, forefoot contact; HO, heel-off; TO, toe-off.

This study was approved by the Ethics Committee of the Zhujiang Hospital of Southern Medical University and written informed consent was provided by each participant. The experimental setting is shown in Figure 1 **.**

### 2.1 Participants

The sample size calculation was based on the COP displacement data reported by Zhang et al. (2017) using GPower 3.1 software (University of Kiel, Kiel, Germany), considering a statistical design of the paired-t test, with a power of 80% and an alfa error of 5%. Based on the calculation, a total of 12 participants were needed to detect differences in the COP variables between different conditions. In case of possible bad trials, we recruited 15 young adults (age: 20.0 ± 1.2 years, height: 172.6 ± 4.5 cm, weight: 63.7 ± 4.6 kg) in this study. All participants were recreational runners who ran at least 10 km per week, and had no history of lower limb injuries in the preceding 6 months, and had a neutral foot type, which was assessed using a 6-item foot posture index (Redmond et al., 2008). They ran an average of 4.7 ± 1.3 times per week, 3.5 ± 1.1 km per run, and 14.7 ± 2.7 km in total. All participants had the same shoe size to avoid the effects of shoe size and for the convenience of the following analyses.

### 2.2 Arch supports

**FIGURE 2:**
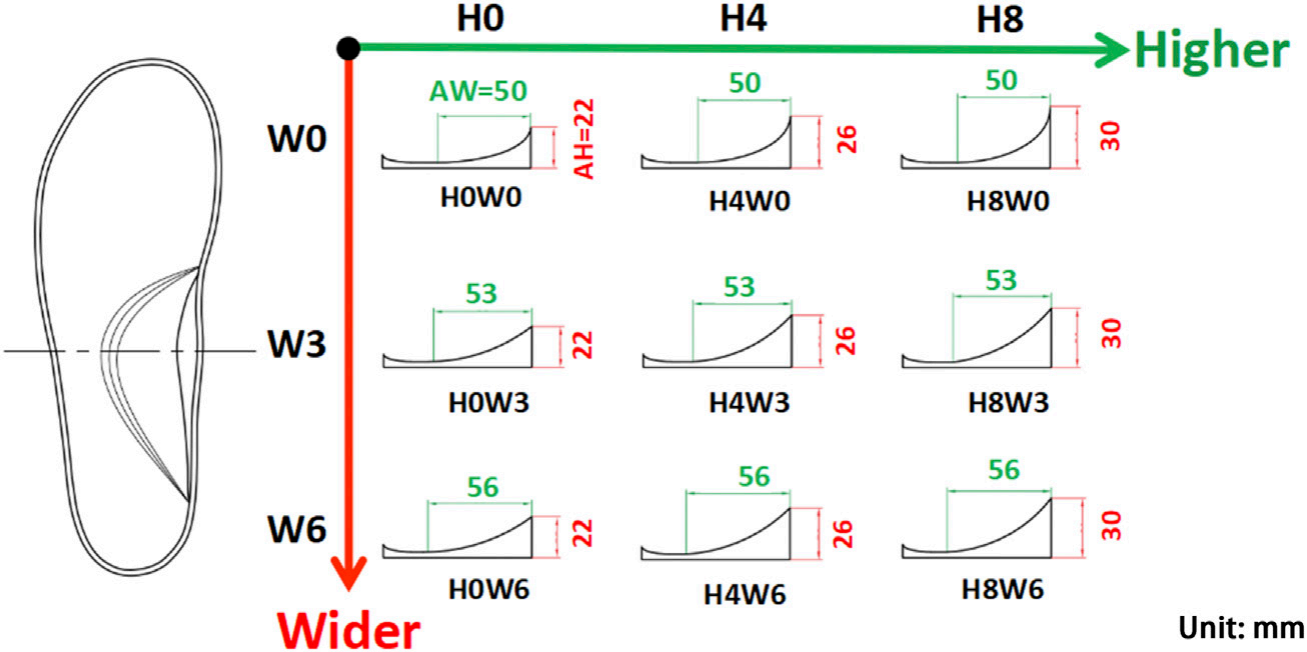
Nine variations of the arch support with three doses in the arch support height (AH: H0, H4, and H8 in mm) and three doses in the arch support width (AW: W0, W3, and W6 in mm).

Insoles with a base arch support (H0W0) were digitally designed in Rhinoceros 3D modeling software (Rhino, Washington DC, United States) based on an averaged 3D scan model of the foot in a standing position (shoe size 9), which was provided by a local running shoe company (Li Ning Sports Ltd., Beijing, China). Variations in the arch support height and width were made upon this base arch support. To set a physiologically reasonable value of each variation, the longitudinal arch (LA) dimensions under different weight-bearing conditions were considered. Using a 3D foot scanning system (Yuandian Technology Co., Ltd., Shenzhen, China) on 30 recreational runners, the LA dimensions under three weight-bearing conditions (sitting, double-leg standing and single-leg standing) were compared. As the weight increased, average alterations of 4 mm in the LA height and 3 mm in the LA width were observed. These values were used to set the arch support parameters. A total of 9 variations of arch supports were designed, with the height and width increments setting at 4 mm and 3 mm, respectively (Figure 2). The insoles were made of EVA with a Shore hardness of 60C.

### 2.3 Data collection

Nine arch supports and a flat insole were put inside neutral running shoes (shoe model: ARBR005-2, Li Ning Sports Ltd., Beijing, China, see Supplementary Figures S1, S2 in Supplementary Material) and tested in a random order. The shoe midsole was made of uniform EVA material with no special structures/designs. Running with the flat insole was defined as the control condition. A 10 min familiarization phase of running was given for each testing condition. Participants were asked to run along a 20 m runway with an integrated 1-m pressure plate (RSscan International, Belgium) at their preferred speeds (3.48 ± 0.01 m/s). The pressure plate has 8,192 resistive sensors (a pixel resolution of 7.62 mm × 5.08 mm) to measure vertical plantar pressure at a frequency of 200 Hz. A laser timer was used to record running speed. A trial was recorded when the running speed was within 5% of the preferred speed of the participant. Five successful steps of each foot were recorded, and data of both feet were pooled together for further analysis. A total of 1,500 time series of the COP trajectory and 1,500 peak pressure images were measured.

### 2.4 Data and statistical analysis

The COP coordinates were exported from the Footscan software and were filtered with a fourth order low pass Butterworth filter at a cutoff frequency of 50 Hz. The end points were padded through a reflection technique, adding 15 data values at start and end of the data series (De Cock et al., 2008). After filtering, the total COP velocity (COPV_total_) was calculated with a simple differentiation and all trials were normalized for the stance time. The stance phase was divided into four sub-phases: initial contact phase (ICP), forefoot contact phase (FFCP), foot flat phase (FFP) and forefoot push off phase (FFPOP) (Chiu et al., 2013b). The ICP is defined as the period from initial foot contact (IFC) to initial metatarsal contact (IMC). The FFCP follows ICP until the entire forefoot contacts the pressure plate. The FFP starts from forefoot contact (FFC) to heel-off (HO). The FFPOP is the period from HO to toe-off (TO).

1D and 2D SPM were performed to assess differences between arch support and control conditions for time series of the COP variables and plantar pressure mapping, respectively. These methods use Random Field Theory to make statistical inferences about continuous 1D/2D data to test where signals may differ in time and space domains (Pataky et al., 2011). The COP trajectory in the medial-lateral direction (COP_ML_) and COPV_total_ were compared between the arch support and control conditions using 1D SPM paired-samples *t*-test. For this comparison, the average data of arch supports with the same heights (H0, H4 and H8) and same widths (W0, W3, and W6) were calculated. The plantar pressure data was normalized by foot progression angle (Keijsers et al., 2009) and then 2D SPM paired t-tests were performed to compare the normalized pressure mapping between the arch support and control conditions. In short, 1,500 peak pressure images were realigned firstly to ensure anatomical consistency. After realignment, a general linear model was employed to estimate the parameters of a temporal model and to derive the appropriate univariate test statistic SPM{t} at each pixel. The SPM{t} were assembled into SPM. Finally, statistical inferences were made on the basis of the SPM and Random Field Theory (Pataky, 2008). To determine the main effect for the arch support height, width, and their interaction, two-way ANOVAs were performed on the peak values of COPV_total_. Where significant effects of the arch supports were found, linear, quadratic and cubic contrasts were tested to determine if there was a linear trend to the effect. For all statistical analysis, *p*-values less than 0.05 were considered statistically significant.

## 3 Results

**FIGURE 3:**
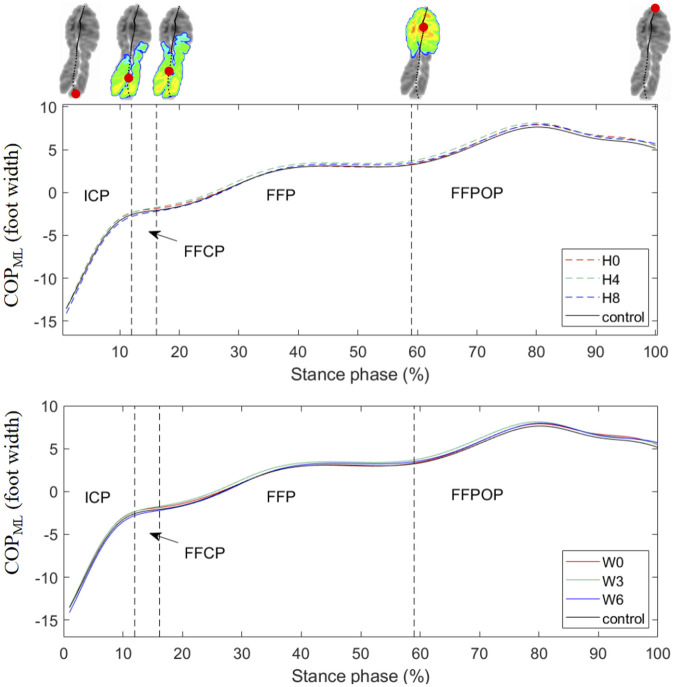
Mean curves of the COP_ML_ coordinates during running. Medial displacements of the COP_ML_ were expressed as positive values. H0, H4, and H8 represented the mean data of arch supports of three different heights; W0, W3, and W6 represented the mean data of arch supports of three different widths. Four subphases are indicated with vertical dash lines on the *x*-axis. ICP, initial contact phase; FFCP, forefoot contact phase; FFP, foot flat phase; FFPOP, forefoot push off phase; IFC, initial foot contact; IMC, initial metatarsal contact; FFC, forefoot contact; HO, heel-off; TO, toe-off.

The mean curves of the COP_ML_ during running are illustrated in Figure 3. The 1D SPM results showed no significant difference in the COP_ML_ trajectory between the arch support and control conditions during the entire stance phase.

**FIGURE 4:**
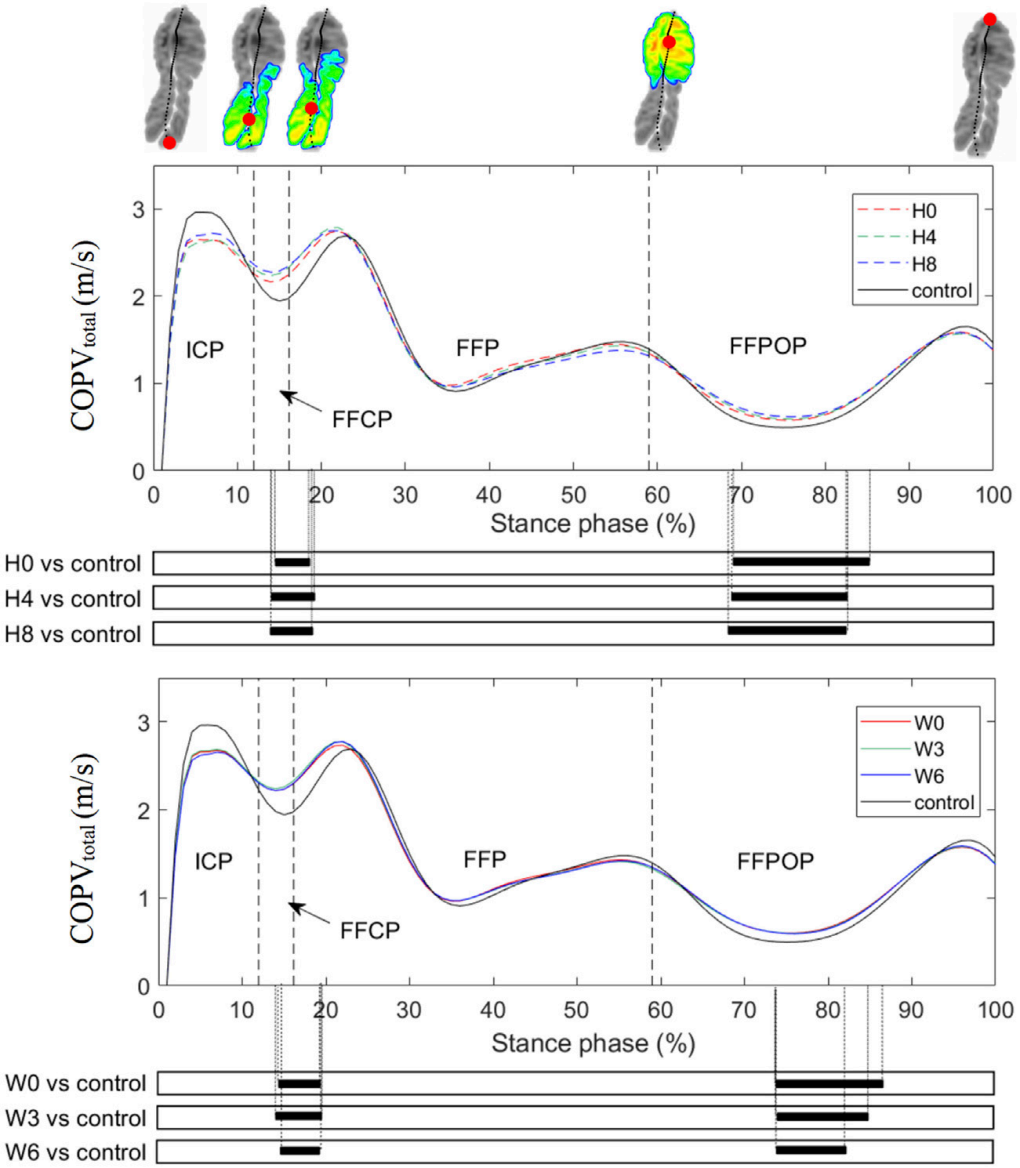
Mean curves of the COPV_total_ during running. H0, H4, and H8 represented the mean data of arch supports of three different heights; W0, W3, and W6 represented the mean data of arch supports of three different widths. Four sub-phases are indicated with vertical lines on the *x*-axis. The black bar below the graph represents the time during which the differences between the groups occurred (*p* < 0.05), what was indicated by the SPM {t} statistics. ICP, initial contact phase; FFCP, forefoot contact phase; FFP, foot flat phase; FFPOP, forefoot push off phase; IFC, initial foot contact; IMC, initial metatarsal contact; FFC, forefoot contact; HO, heel-off; TO, toe-off.

Mean COPV_total_ curves and the SPM results are illustrated in Figure 4, and comparisons in the anterior-posterior COP velocities (COPV_AP_) and medial-lateral COP velocities (COPV_ML_) are shown in Supplementary Figures S3, S4 in Supplementary Material. The COPV_total_ curve showed a pattern of four peaks and three valleys during all shod running. After heel strike, the COPV_total_ increased rapidly to the first peak and dropped to the first valley before forefoot flat. Then the COPV_total_ fluctuated quickly to reach the second peak and valley and slowly up to the third peak before heel-off. During the FFPOP, the COPV_total_ dropped to the third valley and then raised to the fourth peak till toe-off. Compared with the control, all arch supports showed significant differences in the COPV_total_ during the FFCP and FFPOP of running. Similar trends were seen in the COPV_AP_. In contrast, no significant differences in the COPV_ML_ were observed between conditions.

Results of two-way ANOVAs examining two design factors (height and width of arch suppor) on the peaks and valleys of the COPV_total_ are shown in Table 1. The height of arch support had a main linear effect on the first valley, third peak and third valley of the COPV_total_, with a higher arch support causing an increase in the first and third COPV_total_ valleys and a decrease in the third COPV_total_ peak. In contrast, the width showed no effects on tested variables, and no interaction effect between two design factors was observed.

**TABLE 1:** Results of tests of within-subject effects from two-way repeated ANOVAs.

Parameter	Effect	F	*p*-value	Best contrast (change% per 4-mm height increase)
1st peak	height	1.204	0.302	Linear (3% reduction)
	width	0.82	0.424	
	height*width	0.239	0.86	
2nd peak	height	1.881	0.17	
	width	2.845	0.066	
	height*width	0.328	0.859	
3rd peak	height	13.542	**0.001**	Linear (6% increase)
	width	1.559	0.219	
	height*width	0.212	0.931	
4th peak	height	1.559	0.219	
	width	0.373	0.69	
	height*width	0.509	0.729	
1st valley	height	11.19	**0.001**	Linear (4% increase)
	width	0.015	0.985	
	height*width	0.446	0.665	
2nd valley	height	0.302	0.675	
	width	0.579	0.564	
	height*width	2.467	0.068	
3rd valley	height	17.994	**0.001**	
	width	2.474	0.093	
	height*width	0.696	0.545	

Bold values show significant differences.

**FIGURE 5:**
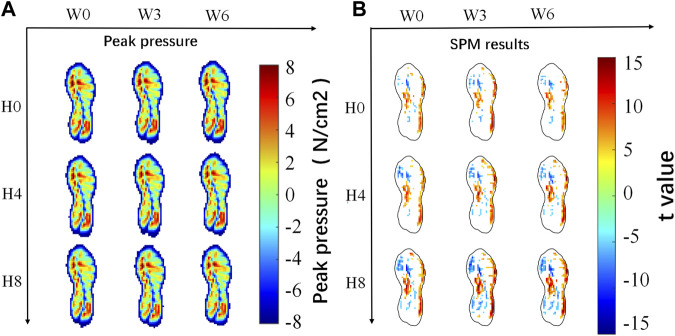
Results of plantar pressure analysis of 9 pairs of different arch supports.

The mean peak pressure mappings of all arch supports are illustrated in Figure 5A. Overall, high peak pressure was located under the medial forefoot and the lateral rearfoot regions in all conditions. The 2D SPM results comparing the arch support and the control insole are shown in Figure 5B, with the blue area showing a significant decrease, and the red area showing a significant increase (*p* < 0.05). A darker color suggests a larger change. It is noticeable that as the arch support height increased, the peak pressure of the medial forefoot and rearfoot decreased, and the pressure of the medial midfoot and the lateral foot increased accordingly. In contrast, there was few visual differences in the plantar pressure mapping when changing the arch support width.

## 4 Discussion

This study investigated the acute effects of nine arch support doses on the 1D and 2D pressure data of running in healthy recreational runners using SPM approaches. The COP_ML_ coordinates and COPV_total_ of different conditions were compared throughout the entirety of stance, and the results showed that a higher arch support was more effective in altering the COPV_total_ than the COP_ML_ coordinates. Furthermore, differences in plantar pressure mapping between the arch support and control conditions were illustrated at the pixel level, with a higher arch support reducing the peak pressure of the medial forefoot and rearfoot. These results could contribute to insole optimization in clinical practice and footwear industry.

Among all COP variables during running, the COP displacements in the medial-lateral direction have been most frequently assessed in literature (Mann et al., 2016), and a more laterally directed COP has been associated with the development of overuse injuries (Ghani Zadeh Hesar et al., 2009). However, the effects of the arch support on this variable were inconsistent across studies, probably due to different test populations, statistical methods, and insole configurations. Compared with a flat insole, Naderi et al. (2019) found that an arch support (peak-height of 25 mm) significantly altered the mean COP_ML_ coordinates of running in a population with medial tibial stress syndrome, while Zhang et al. (2022) found that using two arch supports (peak-height of 20 mm and 24 mm) had little effects on the COP_ML_ trajectory of running in a population with symptomatic pronated feet. The results of the current study (arch support peak-height of 22–30 mm) are in line with the latter one, and further showed that changing neither the height nor the width of the arch support failed to alter the COP_ML_ trajectory during running. Although a medial-lateral COP shift has been used to indicate a load transfer between the medial and lateral foot regions, an absence of such shift may not necessarily suggest that the plantar pressure distribution remained unchanged. This can be verified with the statistical differences in the pressure mapping of this study.

Compared to the COP_ML_ coordinates, our results suggested that the COP velocity was more sensitive to subtle modifications of arch supports. The COPV_total_ could partially reflect the progression velocity of the body center of mass over the foot during the stance phase, providing insights for dynamic foot function. Compared to the flat insole, the arch support significantly increased the COPV_total_ during the FFCP and FFPOP of running (Figure 4), affecting the weight shift and progression of gait (Perry and Burnfield, 2010). The arch support has been shown to increase the propulsive force of running (Ng et al., 2021), and this may explain why the COP advanced more rapidly after heel-off. As one prospective study showed that runners who developed overuse injuries had a lower COP velocity at forefoot flat event than healthy controls during running (Ghani Zadeh Hesar et al., 2009), using an arch support may benefit runners at risk. Furthermore, the arch support may allow the foot to supinate more easily, which would increase the arch stiffness to enable a faster forward propulsion (Kelly et al., 2015). The effect of a faster weight shift and propulsion caused by arch supports on reducing injury risks requires further investigation in the future.

Among all COP velocity variables, peak values of the COPV_total_ contain critical information to evaluate shock absorption, weight shift and propulsion of running. Our results showed a quadruple peak pattern of the COPV_total_ curve during all shod running trials, while the third peak that occurred before heel-off was absent during barefoot walking (Cornwall and McPoil, 2000) and running (De Cock et al., 2008). A rapid initial foot pronation after strike can be speculated from the large value of the first COPV_total_ peak, which enables shock absorption (Perry and Burnfield, 2010). This peak value (approximately 3 m/s) was considerably larger than that of barefoot running (approximately 1 m/s) (De Cock et al., 2008), which might be due to the cushioning property of modern running shoes (Malisoux et al., 2020). In contrast, the second COPV_total_ peak, indicating a fast weight shift from the rearfoot to the forefoot, was comparable with that of the barefoot condition. After heel-off, the COPV_total_ dropped to a plateau, where the third COPV_total_ valley occurred, to prepare propulsion. As COP variables can be easily measured and calculated with a pressure measuring system, the peak COPV_total_ may serve as an important measure of foot function in both clinical and footwear industrial settings.

By changing doses of forefoot and rearfoot wedges, previous studies have established linear dose-response relationships between insole doses and peak/mean biomechanical variables during walking (Telfer et al., 2013a; Telfer et al., 2013b) and running (Zhang et al., 2022). Similarly, the current study examined nine arch support doses, and the statistical results partially validated our hypothesis, with the arch support height having a linear main effect on the COPV_total_ peak values, while its width showing little effect and having no interaction effect with the height. More specifically, as the arch support height increased 4 mm, 6% and 4% increase in the first and third valleys of the COPV_total_ were found respectively. As illustrated above, a faster COP velocity during the FFCP and FFPOP may benefit runners, but how fast is optimal has yet to be determined. A higher arch support also slightly decreased the third COPV_total_ peak, which may be because that a higher arch support would increase the contact with the midfoot region during running (Zhao et al., 2021), slowing down the COP velocity before heel-off. Previous studies have shown that the COP velocity during walking served as an indicator for foot mobility and function during the early healing phase after calcaneal fractures (Klopfer-Kramer et al., 2022). The COP velocity in patients with first metatarsophalangeal joint osteoarthritis (Menz et al., 2018) and posterior tibial tendon dysfunction (Wang et al., 2022) was significantly slower than that in healthy individuals during walking. Therefore, these linear dose-response relationships may help future prescription of insoles for runners.

The 2D SPM results showed that a higher arch support reduced the peak plantar pressure of the medial forefoot and rearfoot. Large peak pressure of those regions should be dealt with caution, as excessive pressure were found there in runners with lower limb injuries (Willems et al., 2007; Naderi et al., 2019). This finding confirmed the prediction results of computational studies using a finite element model (Cheung and Zhang, 2008; Peng et al., 2022). And it was partially in agreement with findings of experimental studies. By dividing the foot into 9 to 10 anatomical regions, Fong et al. (2020) found that using an arch support reduced the rearfoot pressure without altering forefoot pressure during running, while Huang et al. (2020) found that the arch support decreased rearfoot pressure and increased the pressure under the second to fourth metatarsals during walking. It needs to be noted that the pressure was recorded by a pressure insole system by Fong et al. and Huang et al. Those studies compared mean pressure values extracted from discrete anatomical regions, while our study used SPM that allows statistical comparisons at the pixel level. It has been suggested that the regional conflation of the former method may produce calculation errors (Pataky et al., 2008). By generating a higher resolution view of pressure distribution comparisons, the current study may provide references for a more precise management of plantar pressure distribution for clinical and industrial purposes in the future.

Several limitations should be noted for this study. Firstly, the participants of this study were young males, and the effect of aging and gender was not considered. Previous studies show that age and gender affect the COP trajectory during walking (Chiu et al., 2013a; Chiu et al., 2013b), and the elderly and females may have different responses to the arch support doses. Secondly, as the participants only wore each insole for a limited time before testing, and it is not clear whether the pressure variables would alter after a longer adaptation. Subsequent studies should examine the long-term effect of using arch supports. Thirdly, only neutral feet were examined in this study. Future studies are required to determine the dose-response relationship between arch supports and pressure variables in other foot types. Fourthly, the insole material used may also have an influence on plantar pressure.

## 5 Conclusion

Changing arch support doses, primarily the height, affected the COP velocities during loading response and propulsion phases of running, as well as redistributed peak plantar pressure at the pixel level. When assessing subtle modifications in the arch support, the COP velocity was a more sensitive variable than COP coordinates. Furthermore, there was a linear dose-response relationship between the arch support height and peak values of the COPV_total_, and a higher arch support was also more effective in reducing peak plantar pressure of the medial forefoot and rearfoot. The findings of this study would provide insights into the mechanisms of insole interventions and provide potential measures for evaluating foot orthotics and footwear.

## Data Availability

The original contributions presented in the study are included in the article/Supplementary Material, further inquiries can be directed to the corresponding author.
