# Supplementary material for: Effects of arch support doses on the center of pressure and pressure distribution of running using statistical parametric mapping
Source: Front Bioeng Biotechnol. 2022 Nov 21;10:1051747. doi: 10.3389/fbioe.2022.1051747 (PMC9719983; doi:10.3389/fbioe.2022.1051747)
Supplement: Supplementary file 1 [file DataSheet1.pdf]

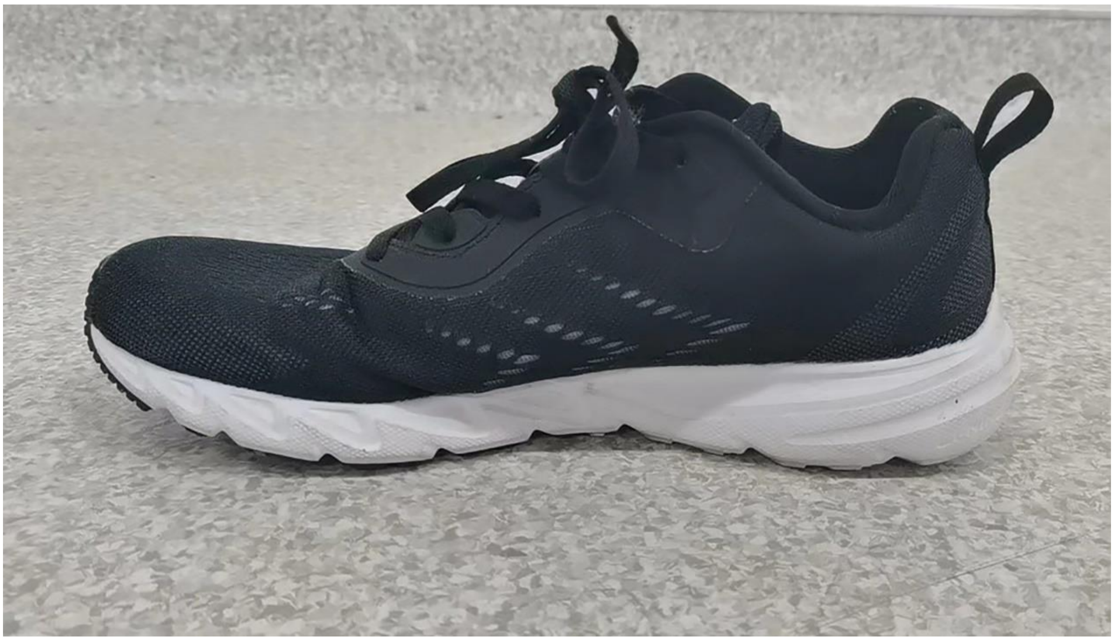

FIGURE S1. Photo from the side view of the shoe (the midsole was made of uniform EVA material)

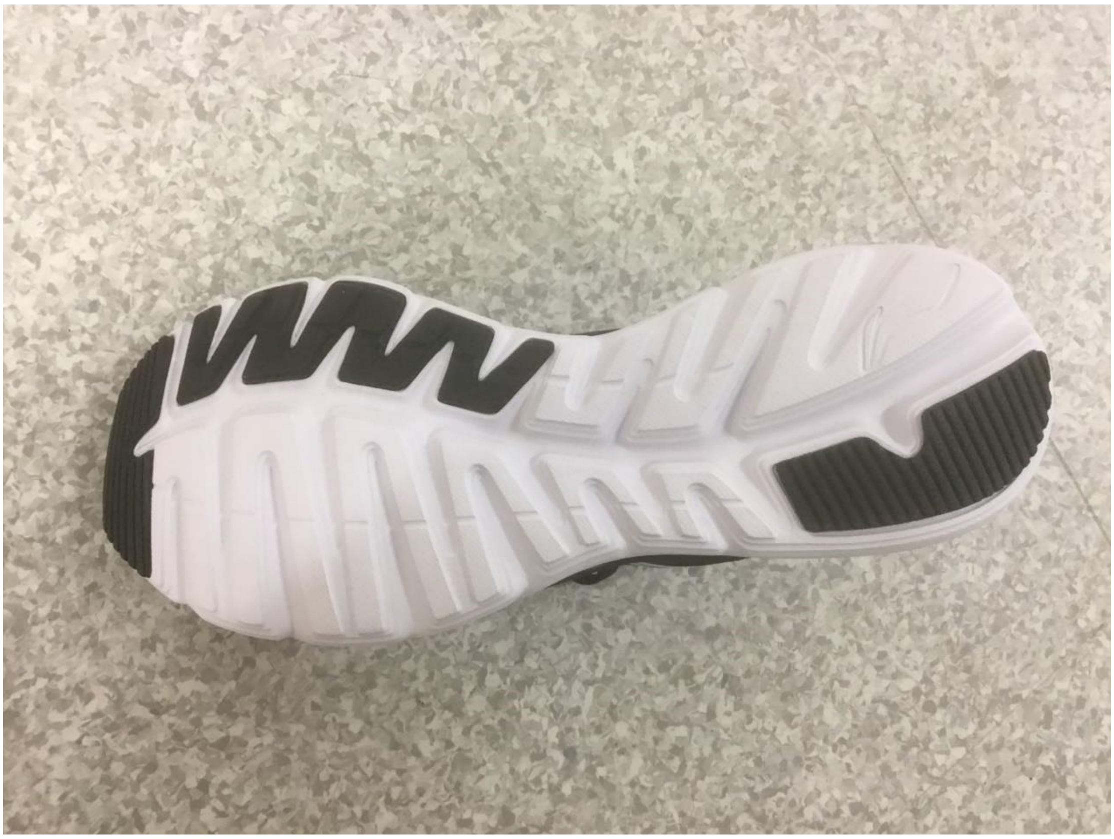

FIGURE S2. Photo from the bottom view of the shoe

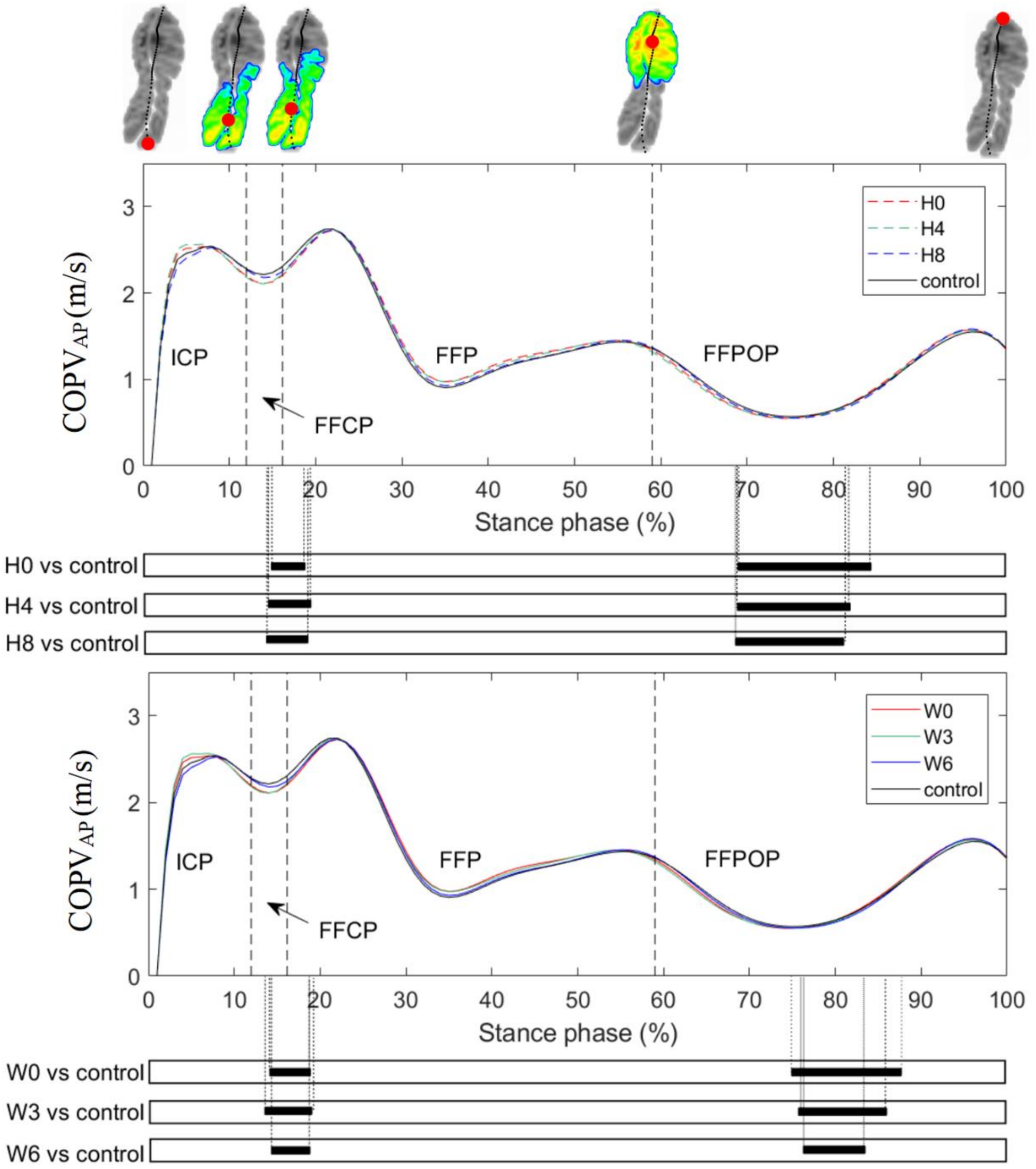

FIGURE S3. Mean curves of the COPV$_{AP}$ during running. H0, H4 and H8 represented the mean data of arch supports of three different heights; W0, W3 and W6 represented the mean data of arch supports of three different widths. Four sub-phases are indicated with vertical lines on the x-axis. The black bar below the graph represents the time during which the differences between the groups occurred ($p < 0.05$), what was indicated by the SPM {t} statistics. ICP: initial contact phase ;FFCP: forefoot contact phase ; FFP: foot flat phase;FFPOP: forefoot push off phase; IFC: initial foot contact; IMC: initial metatarsal contact; FFC: forefoot contact (FFC); HO: heel-off; TO: toe-off.

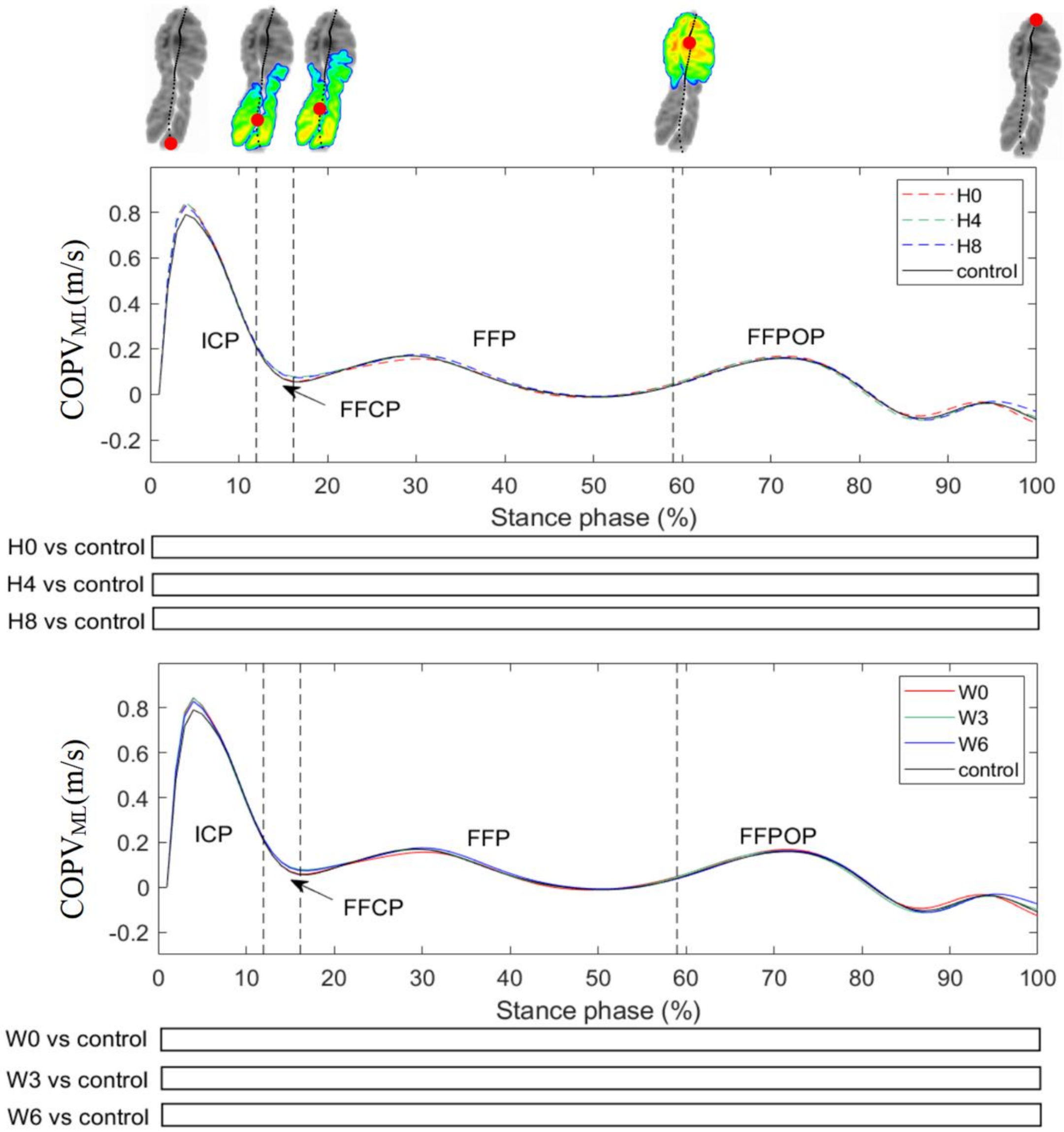

FIGURE S4. Mean curves of the COPV$_{ML}$ during running. H0, H4 and H8 represented the mean data of arch supports of three different heights; W0, W3 and W6 represented the mean data of arch supports of three different widths. Four sub-phases are indicated with vertical lines on the x-axis. The black bar below the graph represents the time during which the differences between the groups occurred ($p < 0.05$), what was indicated by the SPM {t} statistics. ICP: initial contact phase ;FFCP: forefoot contact phase ; FFP: foot flat phase;FFPOP: forefoot push off phase; IFC: initial foot contact; IMC: initial metatarsal contact; FFC: forefoot contact (FFC); HO: heel-off; TO: toe-off.
